# Haplotype hitchhiking promotes trait coselection in *Brassica napus*

**DOI:** 10.1111/pbi.12521

**Published:** 2016-01-23

**Authors:** Lunwen Qian, Wei Qian, Rod J. Snowdon

**Affiliations:** ^1^ Department of Plant BreedingIFZ Research Centre for Biosystems, Land Use and NutritionJustus Liebig UniversityGiessenGermany; ^2^ College of Agronomy and BiotechnologySouthwest UniversityChongqingChina

**Keywords:** linkage disequilibrium, LD, haplogroups, selection, oilseed rape, breeding

## Abstract

Local haplotype patterns surrounding densely spaced DNA markers with significant trait associations can reveal information on selective sweeps and genome diversity associated with important crop traits. Relationships between haplotype and phenotype diversity, coupled with analysis of gene content in conserved haplotype blocks, can provide insight into coselection for nonrelated traits. We performed genome‐wide analysis of haplotypes associated with the important physiological and agronomic traits leaf chlorophyll and seed glucosinolate content, respectively, in the major oilseed crop species *Brassica napus*. A locus on chromosome A01 showed opposite effects on leaf chlorophyll content and seed glucosinolate content, attributed to strong linkage disequilibrium (LD) between orthologues of the chlorophyll biosynthesis genes *EARLY LIGHT‐INDUCED PROTEIN* and *CHLOROPHYLL SYNTHASE*, and the glucosinolate synthesis gene *ATP SULFURYLASE 1*. Another conserved haplotype block, on chromosome A02, contained a number of chlorophyll‐related genes in LD with orthologues of the key glucosinolate biosynthesis genes *METHYLTHIOALKYMALATE SYNTHASE‐LIKE 1* and *3*. Multigene haplogroups were found to have a significantly greater contribution to variation for chlorophyll content than haplotypes for any single gene, suggesting positive effects of additive locus accumulation. Detailed reanalysis of population substructure revealed a clade of ten related accessions exhibiting high leaf chlorophyll and low seed glucosinolate content. These accessions each carried one of the above‐mentioned haplotypes from A01 or A02, generally in combination with further chlorophyll‐associated haplotypes from chromosomes A05 and/or C05. The phenotypic rather than pleiotropic correlations between leaf chlorophyll content index and seed GSL suggest that LD may have led to inadvertent coselection for these two traits.

## Introduction

Chlorophyll is a green photosynthetic pigment with which plant chloroplasts generate energy from light. Leaf chlorophyll content relates to photosynthetic capacity and is thus one of the important physiological traits influencing crop yield (Czyczyło‐Mysza *et al*., 2013; Wang *et al*., 2008). On the other hand, the presence of chlorophyll in mature seeds can be an undesirable trait that can affect seed maturation, seed oil quality, meal quality and germination (Delmas *et al*., 2013). Chlorophyll pigments remaining in processed vegetable oils are also associated with increased oxidation causing rancidity (Tautorus and Low, 1993) and difficulties in hydrogenation (Abraham and Deman, 1986). Recent studies suggested a previously unknown relationship between chlorophyll content and metabolism of glucosinolates, a class of sulphurous secondary metabolites expressed in vegetative and generative tissues throughout almost all plants of the *Brassicales*. For example, a total of 11 light‐harvesting chlorophyll (LHC) protein complex proteins (including eight LHCB and three LHCA proteins) were identified to be down‐regulated in RNAi lines that suppressed molecular networks controlling glucosinolate metabolism in Arabidopsis (Chen *et al*., 2012). Yang and Zhu (2009) reported a potential negative correlation between chlorophyll and glucosinolate content under abiotic stresses in cabbage plants. These results suggest potential genetic and/or metabolic associations between chlorophyll content and glucosinolate metabolism in *Brassica* crops. A molecular basis for such a pleiotropic relationship might be associated with the role of plastids in sulphate reduction and cysteine/methionine synthesis in the chloroplasts, providing sulphuric amino acids that are exported across the chloroplast membrane and play an important role as precursors for glucosinolate synthesis (Takahashi *et al*., 2011). Such a relationship is potentially relevant both evolutionarily and agronomically. Leaf glucosinolates have demonstrated positive nutritional value in vegetable *Brassica* crops (Murillo and Mehta, 2001) and play an important role, either antagonistically or mutualistically, in plant interactions with insect pests. On the other hand, glucosinolates are highly undesirable in *Brassica* oilseed meals fed to livestock (Friedt and Snowdon, 2010). The huge global importance of oilseed rape and canola (*B. napus*), the world's second most important oilseed crop, imparts enormous agroeconomic relevance on these compounds.

Genome‐wide association studies (GWAS) examine cotransmission of phenotypes with genetic markers, normally based on linkage disequilibrium (LD) analysis in genetically diverse populations using panels of markers spanning the entire genome at high density. Besides providing high mapping resolution by incorporating historical recombination events, LD analyses can also provide important insight into the history of both natural and artificial selection (breeding) and give valuable guidance to breeders seeking to diversify crop gene pools.

Natural and artificial selection can cause conservation of haplotype blocks, comprising specific combinations of nucleotides on the same chromosome, in genome regions carrying genes under positive or negative selection. Haplotypes can therefore provide more information than any single SNP regarding the complex relationship between DNA variation and quantitative phenotypes (Stephens *et al*., 2001). Elucidating the evolutionary relationships among local haplotypes can further improve the detection power of GWAS scans (Buntjer *et al*., 2005). For example, detailed analysis of LD surrounding major QTL revealed strong signatures of artificial selection associated with important traits in different breeding pools of rapeseed and bread wheat (Qian *et al*., 2014; Voss‐Fels and Snowdon, 2015; Voss‐Fels *et al*., 2015). In such cases, haplotypes reveal the extent to which genetic variation in a given chromosome region is described by clustering markers. Comparing haplotype diversity can help to understand the effects of natural and artificial selection on genome‐scale and single‐gene variation, as shown recently in grapevine (Fernandez *et al*., 2014), maize (Yang *et al*., 2013) and Arabidposis (Li *et al*., 2014). New high‐density genome screening tools provide an unprecedented level of insight into local LD patterns in even complex crop genomes (Edwards *et al*., 2013; Voss‐Fels and Snowdon, 2015). For example, strongly selected haplotype patterns detected in high‐density population genomic studies have been associated with domestication, adaptation and breeding in sorghum (Mace *et al*., 2013), rapeseed (Qian *et al*., 2014) and bread wheat (Voss‐Fels *et al*., 2015), respectively.

Evolutionary selection pressures frequently act on entire pathways or their functional subnetworks. Multiple interacting genes may change in the same fitness direction, at a similar evolutionary rate and across the same timescale, to achieve a common phenotypic outcome. Associations in evolutionary patterns may therefore simply reflect parallel selection of different genes in the same pathway with shared functionality. On the other hand, artificial selection in plant breeding targets recombinations surrounding chromosome regions that carry variants conferring traits of agricultural or economic interest. Different traits that interact via molecular networks may be unintentionally coselected due to pleiotropy, whereas traits controlled by distinct networks may be coselected via LD between functionally independent genes. Haplotype blocks can provide powerful insight into the causes of correlations between different, quantitative trait phenotypes and their associations with responsible genes within haplotype regions.

In this study, we used a high‐density SNP genotyping array to identify haplotype blocks associated with leaf chlorophyll content index (CCI) and seed glucosinolate content in a diverse *B. napus* population. Gene content within haplotype blocks for these two traits suggests they have been coselected during breeding of high‐yielding, high‐quality, modern oilseed rape cultivars. Our results suggest their interrelationship in *B. napus* can be explained by hitchhiking selection due to LD between functionally nonrelated genes.

## Results

### Variation and correlations for leaf chlorophyll and seed glucosinolate content

**Figure 1:**
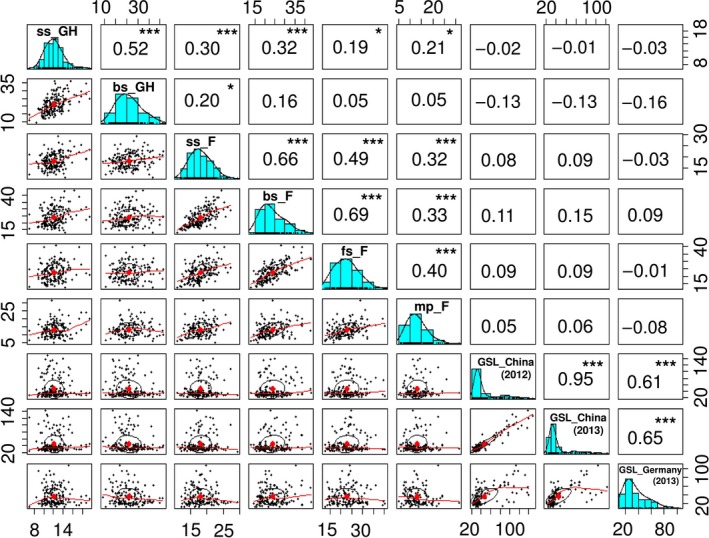
Correlation coefficients and frequency distributions for chlorophyll content index and GSL in 203 Chinese semi‐winter rapeseed accessions. ss_GH: chlorophyll content index in seedling stage (glasshouse experiments, 2012); bs_GH: chlorophyll content index in bolting stage (glasshouse experiments, 2012); ss_F: chlorophyll content index in seedling stage (Field, 2013); bs_F: chlorophyll content index in bolting stage (Field, 2013); fs_F: chlorophyll content index in flowering stage (field experiments, 2013); mp_F: chlorophyll content index in mature period (Field, 2013); GSL: glucosinolate content (*P ≤ 0.05, **P ≤ 0.01, ***P ≤ 0.001).

Broad variation was observed in leaf CCI across the diversity panel, in different field and glasshouse environments, and across different plant developmental stages. Highly significant (*P *< 0.001) positive correlations were seen between different developmental stages in the same location (Figure 1). Weaker but nevertheless significant correlations were measured between field and glasshouse, while leaf CCI at bolting stage in the glasshouse was not significantly correlated with bolting stage, flowering stage and mature period in the field. A low heritability of *H* ^2^ = 0.24 was calculated for leaf CCI, reflecting the strong G*E interaction (Table S1). As expected from previous findings, seed GSL was highly heritable (*H* ^2^ = 0.86) (Table S1). Correspondingly, highly significant positive correlations (*P *< 0.001) were found among the GSL values from the diversity panel among the three different environments (Figure 1).

### Homoeologous haplotype blocks containing chlorophyll‐related genes associated with leaf CCI

Manhattan plots and quantile–quantile plots describing significant SNP associations for leaf CCI in glasshouse and field experiments are shown in Figure S1. A total of 35 and 32 SNPs distributed throughout the genome were detected with the significance threshold of –log10^(*P*)^ = 4 using the glasshouse and field data, respectively. Associations to CCI that could be corroborated in multiple environments and tissues were selected as candidate loci for further investigation. Candidate regions containing SNPs associated with leaf CCI were investigated at high resolution by assaying haplotype blocks (*r* ^2^  > 0.65) in flanking chromosome segments. Details of SNPs and candidate genes in haplotype blocks with significant associations to leaf CCI are provided in Figure S2 and Table S2.

**Figure 2:**
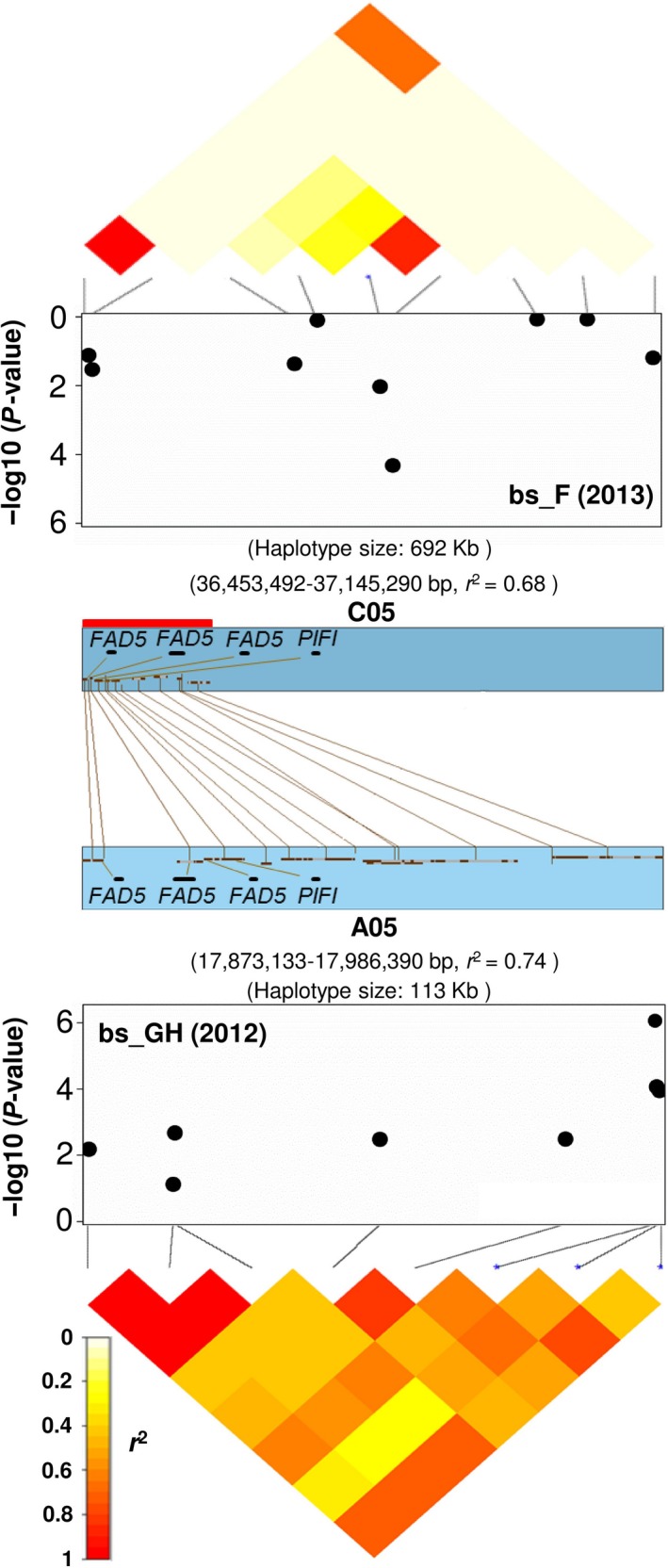
Association mapping for leaf chlorophyll content index at bolting stage in glasshouse (bs_GH) and field (bs_F) to homoeologous haplotype regions on of *Brassica napus* chromosomes A05 (17 873 133–17 986 390 bp) and C05 (36 453 492–37 145 290 bp). The heatmaps span the SNP markers that show linkage disequilibrium with the most strongly associated SNPs. Three *B. napus* orthologues of the gene *FAD5* and one of *PIFI* are located in each of the homoeologous segments (indicated by the red bar).

Two SNPs (Bn‐A05‐p19777231 and Bn‐A05‐p19777547 with *P* = 8.61 × 10^−7^ and 8.59 × 10^−5^, respectively) with significant associations to CCI were located in a 113 kb haplotype region on chromosome A05 (position 17 873 133–17 986 390 bp; *r* ^2^ = 0.74) (Figure 2; Table S3). As shown in Figure 2, comparative analysis via synteny alignments revealed that this region is homologous with a 692 kb haplotype block on chromosome C05 (position 36 453 492–36 605 598 bp; *r* ^2^ = 0.68) that also showed significant associations to leaf CCI (Table S3). Both homologous regions contain multiple *B. napus* orthologues of the *Arabidopsis* chloroplast membrane gene *PALMITOYL‐MONOGALACTOSYLDIACYLGLYCEROL DELTA‐7 DESATURASE* (*FAD5*; BnaA05g23670D, BnaA05g23680D, BnaA05g23690D, BnaC05g37420D, BnaC05g37450D and BnaC05g37460D), along with two copies of the photosynthesis gene *POST‐ILLUMINATION CHLOROPHYLL FLUORESCENCE INCREASE PROTEIN* (*PIFI*; BnaA05g23700D and BnaC05g37470D) (Table S3). Three and nine haplogroups were observed for the A05 and C05 haplotype regions, respectively. Two haplogroups, A05_Hap2 and C05_Hap5, were found to exhibit higher CCI than the remaining two and eight haplogroups on chromosomes A05 and C05, respectively (*t*‐test and mean values; Figure S3; Table S3).

### Chlorophyll‐associated haplotypes carry chloroplast membrane protein genes

**Figure 3:**
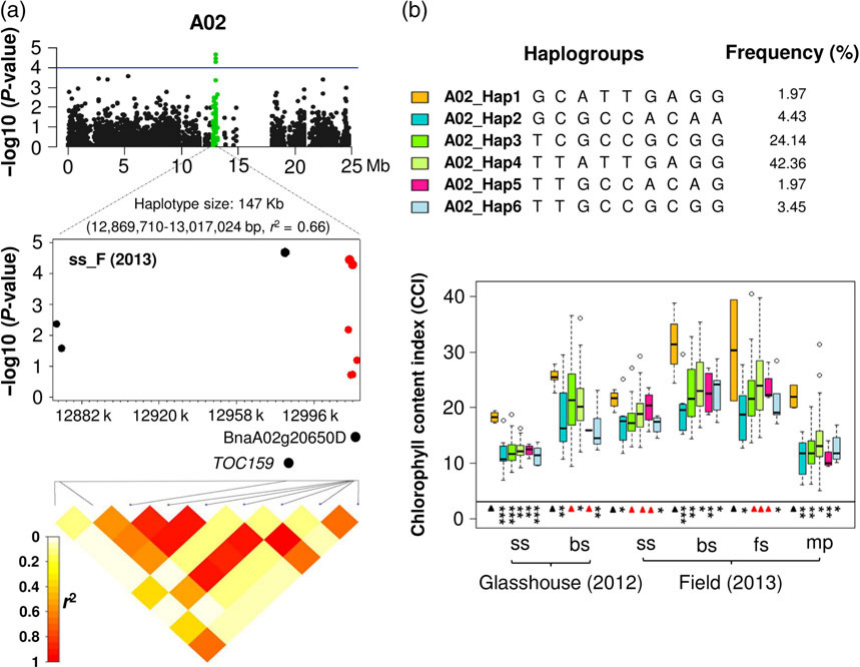
Association mapping for leaf chlorophyll content index (CCI) on chromosome A02 in 203 Chinese semi‐winter rapeseed accessions. (a) Green plots show a 12 869 710–13 017 024 bp haplotype region significantly associated with leaf CCI. The blue horizontal line indicates a threshold of genome‐wide significance at a *P* value of 1.0 × 10^−4^. The heatmaps span the SNP markers that show linkage disequilibrium (LD) with the most strongly associated SNPs. Positions of *Brassica napus* orthologues of the *Arabidopsis* genes *TOC159* (BnaA02g20610D) and a chloroplast thylakoid lumen protein (BnaA02g20650D) are marked. The six SNPs labelled in red are located within the gene BnaA02g20650D. (b) Boxplots showing leaf CCI values for six haplogroups with frequency >0.01. Symbols show significant differences of haplogroups compared with A02_Hap1: *P ≤ 0.05, **P ≤ 0.01, ***P ≤ 0.001; Red triangles: not significant (*P *> 0.05).

Three SNPs (Bn‐A05‐p9118240, Bn‐A05‐p9079762 and Bn‐A05‐p9078313) in a 147 kb haplotype block (*r* ^2^ = 0.66), from 12 869 710 to 13 017 024 bp on chromosome A02, showed significant associations (*P* = 2.088 × 10^−5^, 3.63 × 10^−5^ and 5.17 × 10^−5^, respectively) to leaf CCI (Fig 3a; Table S4). The haplotype block containing this gene also contains *B. napus* orthologues of the Arabidopsis genes *TRANSLOCON AT THE OUTER ENVELOPE MEMBRANE OF CHLOROPLASTS 159* (*TOC159*; BnaA02g20610D) and AT4G02530 (BnaA02g20650D). *TOC159 e*ncodes an essential component of the TOC‐protein complex, responsible for recognition and translocation of photosynthetically active proteins through the chloroplast envelope membrane (Bauer *et al*., 2000), while BnaA02g20650D encodes a chloroplast thylakoid lumen protein involved in photosynthesis and chlorophyll biosynthesis (Ferro *et al*., 2010; Friso *et al*., 2004). By comparing leaf CCI phenotypes of the haplogroups for this haplotype block, we found that the haplogroup A02_Hap1 was associated with higher chlorophyll levels than the other five haplogroups (*t*‐test and mean value; Figure 3b; Table S4). A02_Hap1 differs strongly differs from all other haplogroups except for A02‐Hap4, which has differences at only the first two bases. Genotypes carrying A02‐Hap4 also have higher median CCI in later developmental stages (Figure 3, Table S4).

**Figure 4:**
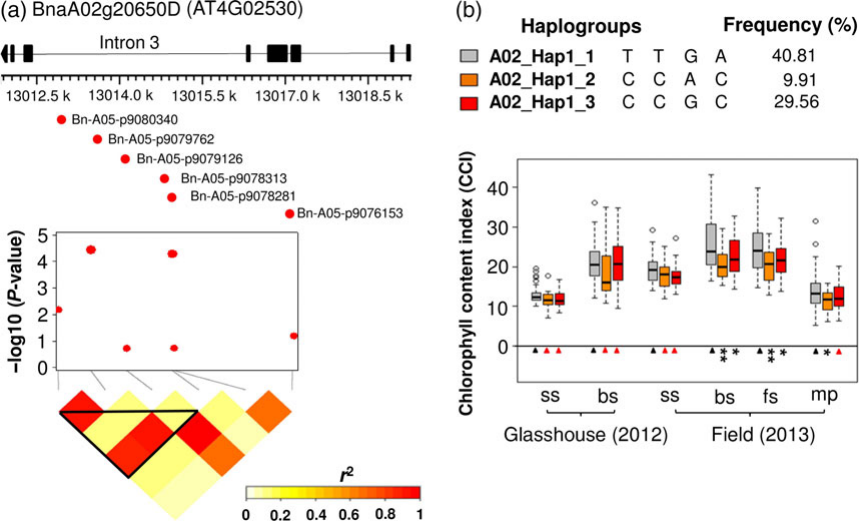
(a) The SNPs Bn‐A05‐p9079762 and Bn‐A05‐p9078313, located in intron 3 of BnaA02g20650D within a 2 kb haplotype block, exhibited significant associations with leaf chlorophyll content index (CCI). (b) Boxplots showing leaf CCI values for three haplogroups with frequency >0.01 within the BnaA02g20650D gene‐haplotype region. Symbols show significant differences of haplogroups compared with A02_Hap1_1: *P ≤ 0.05, **P ≤ 0.01, ***P ≤ 0.001; Red triangles: not significant (*P *> 0.05).

**Figure 5:**
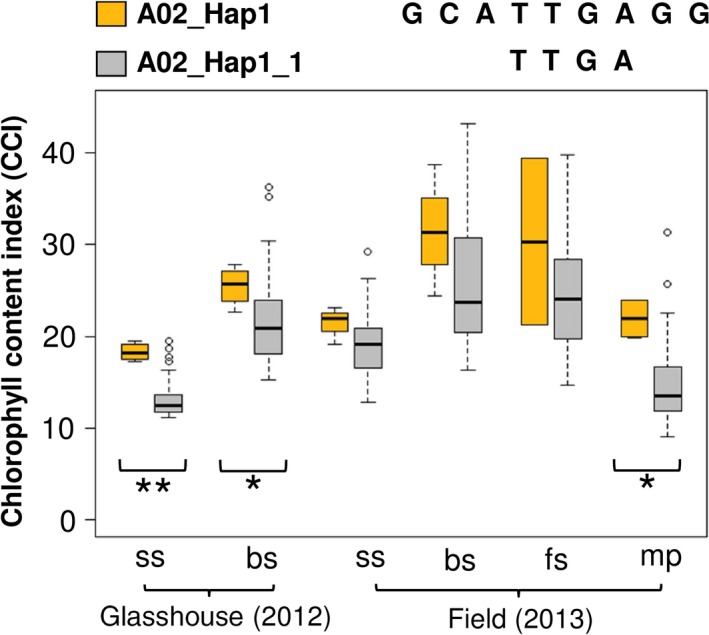
Boxplots showing phenotypic values for leaf chlorophyll content index (CCI) related to two haplogroups. Comparison between the haplogroups show higher leaf CCI in A02_Hap1 than A02_Hap1_1 (*P ≤ 0.05, **P ≤ 0.01).

In addition, six SNPs in this haplotype region were located within the gene BnaA02g20650D. Two of these SNPs (Bn‐A05‐p9079762 and Bn‐A05‐p9078313), located within a 2 kb haplotype block inside intron 3 of BnaA02g20650D, exhibited significant associations with leaf CCI (Figure 4a; Table S5). We used t‐test and mean values to compare phenotype values for three haplogroups identified in the 2 kb haplotype block. Haplogroup A02_Hap1_1 was found to have higher leaf CCI than other two haplogroups (Figure 4b; Table S5). Comparison of A02_Hap1_1 with A02_Hap1 showed that A02_Hap1 (including both BnaA02g20650D and BnaA02g20610D) has higher leaf CCI than A02_Hap1_1 (comprising only BnaA02g20650D) (Figure 5). This result suggests that the multigene haplogroup has a significantly greater contribution to higher leaf chlorophyll content than the single gene haplogroup.

### Haplotype conservation associates with coselection for leaf CCI and seed GSL

In a number of CCI‐associated haplotype blocks, we observed strong LD between genes involved in chlorophyll biosynthesis, photosynthesis or chloroplast membrane fatty acid synthesis, and further genes implicated in GSL synthesis. We therefore mapped SNP‐trait associations for seed GSL, using phenotype data from three different environments, to test whether the same haplotype block regions are also associated with GSL. A genome‐wide significance threshold of –log^10(*P*)^ = 4.4 was applied to determine SNPs with significant associations to GSL.

**Figure 6:**
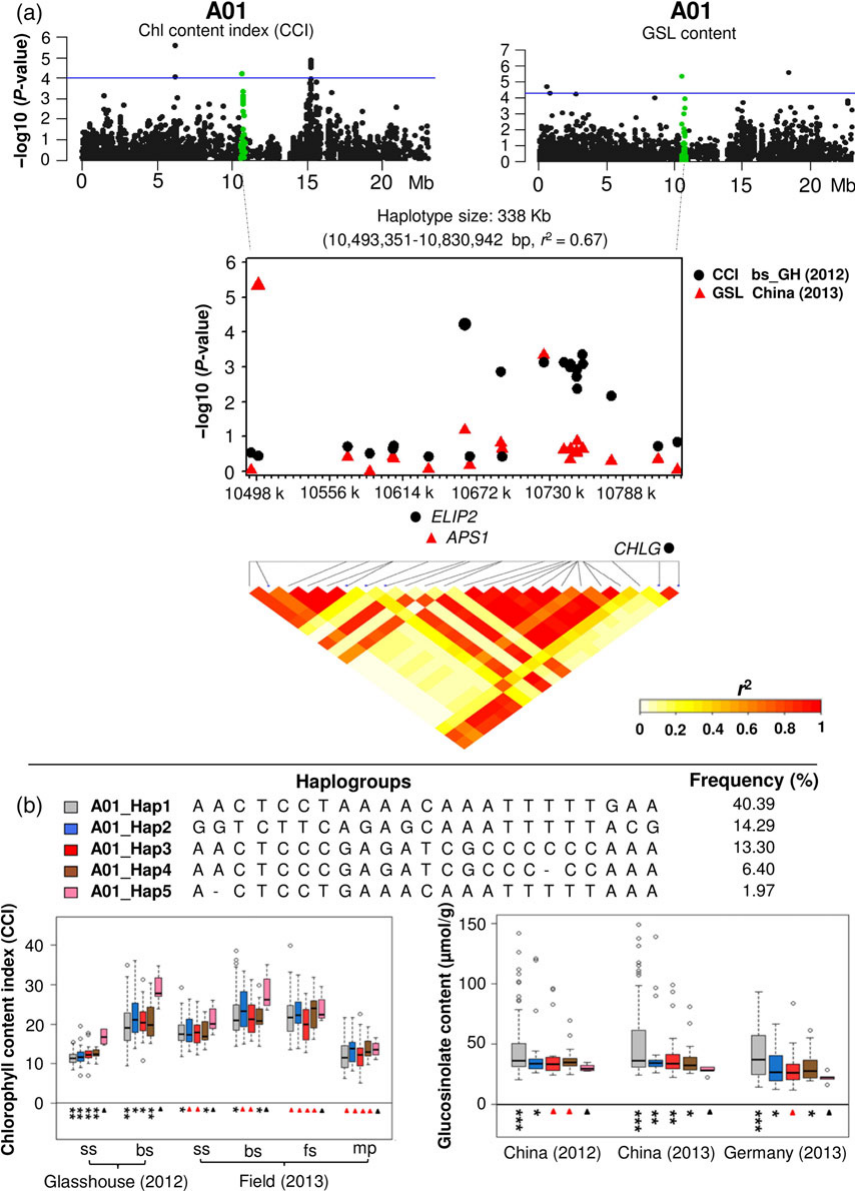
Association mapping for leaf chlorophyll content index (CCI) and seed GSL on chromosome A01. Green plots represent a haplotype region (36 453 492–37 145 290 bp) significantly associated with leaf CCI and seed GSL, respectively. The blue horizontal line indicates a threshold of genome‐wide significance at a *P* value of 1.0 × 10^−4^ and 4.5 × 10^−5^ for CCI and GSL, respectively. The SNPs with highest p values for each trait were used to define a haplotype region with strong linkage disequilibrium (LD) to the traits, containing *Brassica napus* orthologues of two Arabidopsis genes related to chlorophyll (*ELIP2* and *CHLG*) and one related to GSL (*APS1*). (b) Five haplogroups with frequency >0.01 were found in this haplotype region. Boxplots show that A01_Hap5 has higher leaf CCI and lower seed GSL than the other four haplogroups. Symbols show significant differences of haplogroups compared with A01_Hap5: *P ≤ 0.05, **;*P* ≤ 0.01, ***P ≤ 0.001; Red triangles: not significant (*P *> 0.05).

Conserved LD blocks containing significant GWAS hits for both seed GSL content and leaf chlorophyll content in chromosome A01 are shown in Figure 6 and Table S6. The SNP markers Bn‐A01‐p12454306 (*P* = 6.00 × 10^−5^) and Bn‐A01‐p12314813 (*P* = 4.57 × 10^−6^) were significantly correlated to CCI and GSL, respectively (Figure 6a; Table S6). These two markers share conserved LD within a 338 kb haplotype block (*r* ^2^ = 0.67) containing *B. napus* orthologues of three chloroplast‐associated *Arabidopsis* genes with functional annotations associated to chlorophyll and glucosinolates, respectively (Figure 6a; Table S6). The genes *EARLY LIGHT‐INDUCED PROTEIN* (*ELIP2*; BnaA01g19110D) and *CHLOROPHYLL SYNTHASE* (*CHLG*: BnaA01g19280D) are involved in photosynthesis via regulation of chlorophyll biosynthesis, whereas *ATP SULFURYLASE 1* (*APS1*; BnaA01g19120D) encodes the first enzyme in the sulphate assimilation pathway and therefore has a potential direct impact on synthesis of sulphuric glucosinolates (Table S6). For this haplotype block, the haplogroup A01_Hap5 showed higher leaf CCI, especially at seedling and bolting stages in the glasshouse experiments (*P* < 0.05/0.01), whereas A01_Hap1 showed lower CCI than the other four haplogroups (*t*‐test and mean value; Figure 6b; Table S6). Conversely, in all three test environments, haplogroup A01_Hap5 showed significantly lower GSL and haplogroup A01_Hap1 significantly higher GSL than the other four haplogroups (*t*‐test and mean value; Figure 6b; Table S6). In almost all cases, the CCI in the glasshouse experiments showed a negative correlation between leaf CCI and seed GSL between haplogroups A01_Hap1 and A01_Hap5, suggesting that this haplotype may be associated with inadvertent coselection for high leaf chlorophyll in breeding materials with low seed GSL.

### Introgressions from winter oilseed rape contribute additively to chlorophyll content

**Figure 7:**
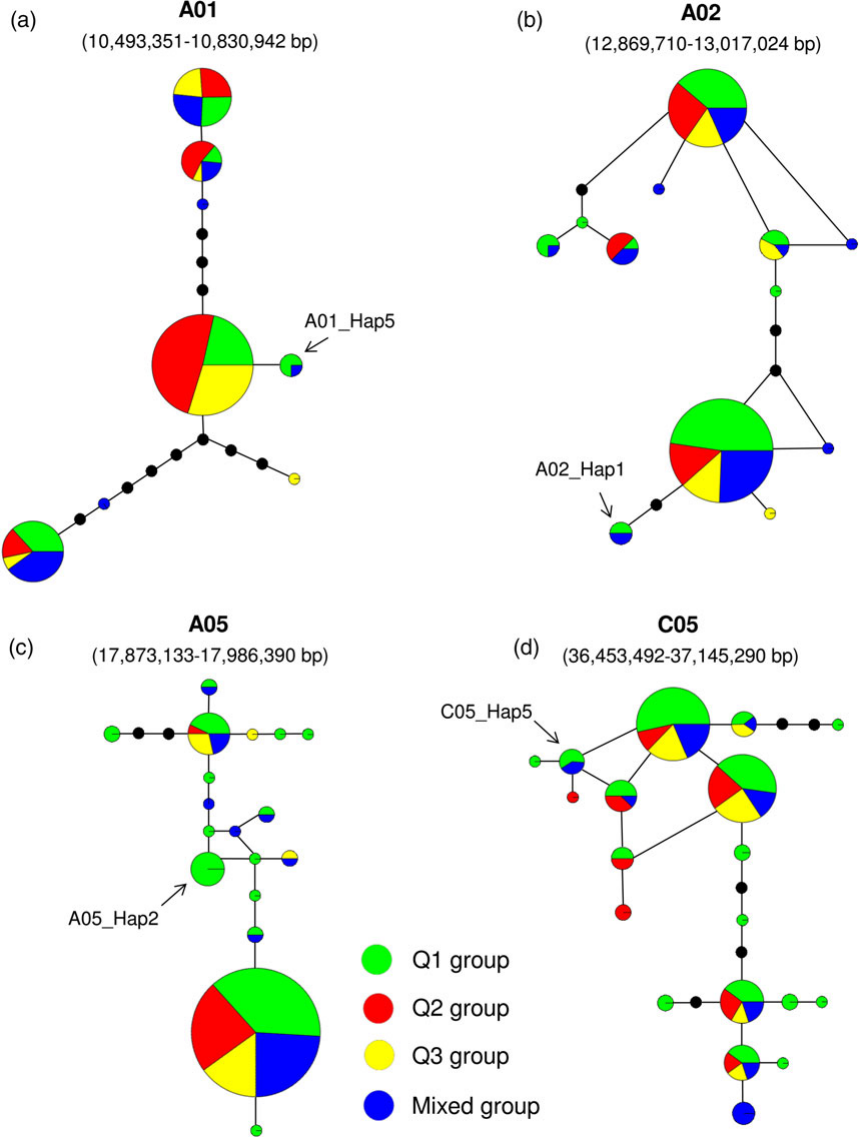
Haplotype networks in the four selected haplotype regions on chromosomes (a) A01 (10 493 351–10 830 942 bp), (b) A02 (12 869 710–13 017 024 bp), (c) A05 (17 873 133–17 986 390 bp) and (d) C05 (36 453 492–37 145 290 bp). Each circle represents a haplogroup, and the size of the circle is proportional to the number of lines within the haplogroup. Colours represent four different subgroups. The arrows indicate the four haplogroups from the Q1 and mixed subpopulations, respectively, which consistently exhibit higher leaf chlorophyll content index.

On chromosomes A01, A02, A05 and C05, the high‐chlorophyll haplogroups A01_Hap5, A02_Hap1, A05_Hap2 and C05_Hap5 were found in 22 of the 203 accessions (Figures 6b, 3b and S3). Haplotype network analysis across these four haplotype regions showed that A01_Hap5, A02_Hap1 and C05_Hap5 correspond to accessions distributed throughout the subpopulations Q1 and ‘mixed’, respectively (Figure 7a, b and d), while accessions carrying A05_Hap2 were found only in Q1 (Figure 7c). Seven of the 22 accessions carried more than one of the high‐chlorophyll haplogroups, and a further 7 also exhibited elevated leaf CCI (Table S7). 11 of these 14 high‐chlorophyll accessions were assigned to subpopulation Q1, which has been shown to be associated with a strong winter rapeseed genetic background Qian *et al*. (2014, Table S7). Collectively, these results suggest that multiple introgressions from winter rapeseed may have elevated chlorophyll content in this group of accessions.

### Haplogroups have additive effects on chlorophyll content

Among the 14 accessions with higher leaf CCI related to haplogroups A01_Hap5, A02_Hap1, A05_Hap2 and C05_Hap5, seven carried combinations of two or three of these haplogroups (designated group A in Table S8). The remaining seven accessions were (designated group B in Table S8) each carry only one of the haplogroups. Comparative phenotype analyses showed the accessions with multiple haplogroups (group A) have higher leaf CCI than those with only one haplogroup (B group; Figure S4; Table S8). This indicates putative additive effects of the selected haplogroups on leaf chlorophyll content.

### Higher chlorophyll content accessions relate to lower GSL

**Figure 8:**
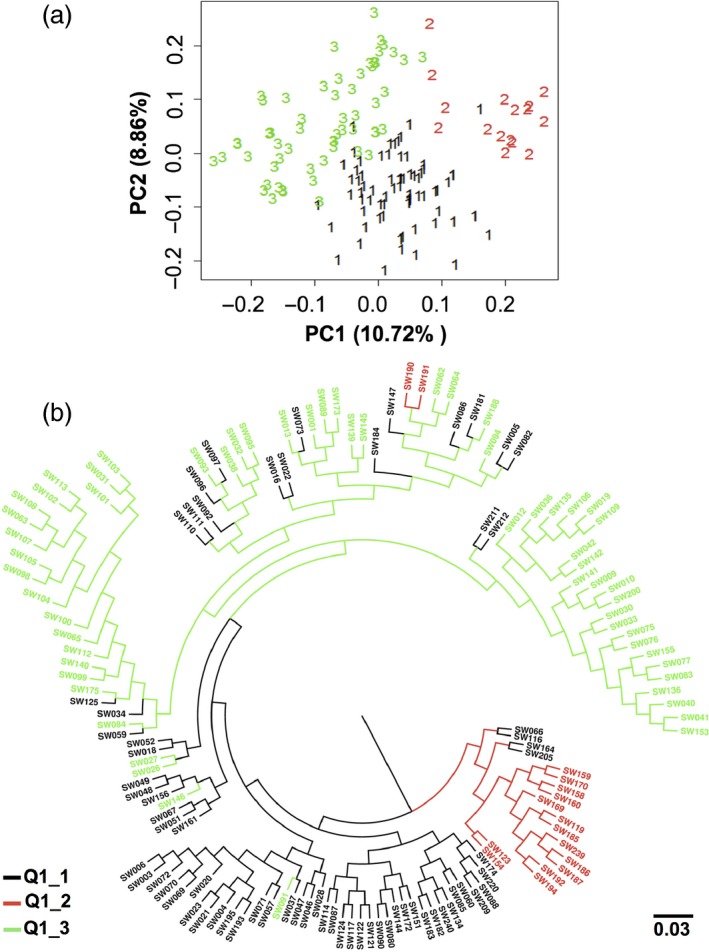
One hundred and forty‐four accessions belonging to the Q1 and mixed subpopulations were reanalysed for detailed population structure by (a) PCA and (b) UPGMA. The 144 accessions clustered into the three clades Q1_1, Q1_2 and Q1_3. The blue dots represent accessions carrying haplogroups, A02_Hap1, A05_Hap2 and C05_Hap5, while the red dots represent accessions carrying haplogroup A01_Hap5.

The reanalysis of detailed substructure in subpopulations Q1 and ‘mixed’ by PCA and UPGMA revealed three clear subgroups (clades Q1_1, Q1_2 and Q1_3), comprising 69, 16 and 59 accessions, respectively (Figure 8a,b; Table S7). The results of the UPGMA tree analysis corresponded with around 84% similarity to the PCA. Haplotype A01_Hap5 (associated with low seed GSL and high leaf CCI) was unique to four accessions distributed across the clade Q1_2. This clade (Figure 8b) also included six accessions with higher leaf CCI associated with the haplotypes A02_Hap1, A05_Hap2 and C05_Hap5, respectively.

Extending the haplotype block A02_Hap1 by only one additional upstream SNP, corresponding to a slightly relaxed LD threshold (*r* ^*2*^ = 0.62; extended haplotype block from 12 869 710 to 13 1667 084 bp), resulted in detection of significant associations with seed GSL (Figure S6; Table S9). As was the case for A01_Hap5, haplotypes associated with lowest GSL overlapped with the haplotypes for high CCI. Within the extended A02_GSL_Hap1 haplotype, we located *B. napus* orthologues of the Arabidopsis glucosinolate biosynthesis genes *METHYLTHIOALKYMALATE SYNTHASE‐LIKE 3* (*MAM3*; BnaA02g20830D) and *MAM 1* (BnaA02g20840D). In Arabidopsis, *MAM1* and *MAM3* play important roles in the biosynthesis of aliphatic glucosinolates (Kroymann *et al*., 2001; Field *et al*., 2004; Textor *et al*., 2007). The very close proximity of these genes (approximately 100 kb) to the CCI‐associated haplotype block confirms the presence of strong LD between putative causal loci for GSL variation and loci with strong effects on CCI. Four accessions belonging to clades Q1_2 and Q1_3 carried the low‐GSL haplotype associated with A02_GSL_Hap1, but not A01_Hap5.

A similar situation to that described for A02_Hap1 was also observed for the haplotype A05_Hap2. Within 200 kb from the 113 kb CCI‐associated haplotype block (*r* ^2^ = 0.74), we found additional genes with a putative function in GSL biosynthesis. This extended haplotype block (*r* ^2^ = 0.41) also showed associations to seed GSL content.

Interestingly, phenotypic comparison among the three clades Q1_1, Q1_2 and Q1_3 revealed higher leaf CCI and lower seed GSL in Q1_2 than in the other two clades (t‐test and mean value; Figure S5; Table S7). This suggests that coselection for these two traits have occurred particularly within clade Q1_2. All individuals belonging to clade Q1_2 carried either A01_Hap5 or A02_Hap1, either alone or in combination with A05_Hap2.

## Discussion

Selection for specific agronomic traits during plant domestication and breeding has strong influences on the genetic diversity and population structure within available gene pools for further crop improvement. High‐density genotyping tools today provide a means for unprecedented insight into patterns of diversity associated with breeding in major crop species (Qian *et al*., 2014; Snowdon *et al*., 2015; Voss‐Fels *et al*., 2015), enabling identification of loci under strong selection and potentially allowing islands of depleted diversity to be addressed by targeted, marker‐assisted introgressions without compromising desirable adaptation, yield or quality traits (Voss‐Fels and Snowdon, 2015).

Besides their negative influence on genetic diversity, strong signatures of selection associated with key traits can also cause coselection of loci with undesirable effects, resulting in what is commonly known as linkage drag. In this study, we present an interesting example in oilseed rape for coselection of increased leaf chlorophyll content along with reduced seed glucosinolate content, two traits with no immediately obvious biological relationship. We demonstrate that introgressions between ecogeographically distinct gene pools resulted in indirect selection of plants with elevated levels of leaf chlorophyll, due to coselection of beneficial haplotypes at four independent haplotype blocks. Within one of these haplotype blocks, a gene involved in chlorophyll synthesis showed almost complete LD with a gene associated with seed glucosinolate content. The relationship between the observed haplotypes and the respective phenotypic behaviour suggests that introgression of this locus during breeding, to reduce seed glucosinolate levels in nutritionally valuable varieties, causes hitchhiking selection resulting in increased chlorophyll content. Elevated chlorophyll may result in improved photosynthetic performance. While this does not necessarily relate *per se* to improved agronomic performance, stay‐green traits associated with increased chlorophyll production, or suppression of chlorophyll degradation, are implicated in improved adaptation of crops to abiotic stresses like water or nutrient deficiency (Thomas and Ougham, 2014).

In this study, a total of nine haplotype blocks were found to harbour significant associations (*r* ^2^ ≥ 0.65) with leaf CCI. Within these regions, we found eighteen genes implicated in chlorophyll synthesis or catabolism, respectively. These included six orthologues of *FAD5*, which was found in Arabidopsis to influence chlorophyll biosynthesis (Heyndrickx and Vandepoele, 2012) and restore leaf chlorophyll content (Heilmann *et al*., 2004), along with two orthologues of the gene *PIFI*, which has an indirect effect on photosynthesis (Gotoh *et al*., 2010). The observations of homoeologous, trait‐associated haplotypes, carrying multiple duplicated genes, provide a further example for gene amplification by polyploidization. In the complex allopolyploid crop *B. napus*, this phenomenon has been shown to influence numerous agronomically important traits (Chalhoub *et al*., 2014).

Additional chlorophyll‐associated haplotypes carried *B. napus* orthologues of the genes *ELIP2* and *CHLG*, which in Arabidopsis are directly involved in chlorophyll biosynthesis (Oster and Rüdiger, 1998; Tzvetkova‐Chevolleau *et al*., 2007), and the chloroplast preprotein transporter protein gene *TOC159*. The latter encodes an essential component of the chloroplast assembly mechanism, which acts as a selective import receptor for preproteins required in chloroplast development (Smith *et al*., 2004). Collectively, the genetic associations we found, involving multiple loci carrying these very different contributors to the photosynthesis apparatus, suggest a broad genetic variation for factors involved in photosynthesis in crop plants. Given the key role of photosynthetic activity in ecogeographical and stress adaptation, it is interesting that multiple loci with positive additive influences on chlorophyll content are coselected in introgressions between different breeding pools in this major crop.

The close genetic relationship among the clade of accessions with simultaneously elevated leaf CCI and low seed GSL suggests that this clade may be the product of artificial selection from a common genetic background. Of the four CCI‐associated haplotypes, two (on chromosomes A01 and A02) carry well‐known glucosinolate biosynthesis genes either within or directly adjacent to the LD block responsible for increase chlorophyll concentration. The third haplotype block, on chromosome A05, is closely linked to genes that have a putative function in GSL synthesis, while its homoeologous locus on chromosome C05 can be expected to carry a corresponding repertoire of homologous genes. Collectively, these examples represent an accumulation of experimental evidence (A02_Hap5 and A02_Hap1) and putative support (A05_Hap2 and C05_Hap5) for a general genetic linkage between loci involved in expression of seed GSL and leaf chlorophyll content in *B. napus*. Leaf CCI and seed GSL exhibit significant associations to different SNP loci that in turn do not appear to show pleiotropic associations to the different traits. The presence of highly plausible positional and functional candidate genes for both traits, and their strong LD to most of the trait‐associated SNPs, provides additional support for the hypothesis that the phenotypic associations we observed are caused by linkage rather than pleiotropy.

The genetic determinants of these two traits appear to differ among the three subclades we identified, however, with overlapping associations among some loci but not others. This suggests that the apparent coselection of chlorophyll content, which in contrast to seed GSL, is not a breeding target in rapeseed, most likely occurred inadvertently (through linkage to loci conferring low seed GSL) rather than intentionally. As such, this represents an interesting example for local enrichment of a trait with specific adaptation potential as an indirect consequence of intensive breeding for seed quality characters.

It is likely that the expression of many genes is jointly controlled by the actions of multiple regulatory alleles. Despite this, GWAS studies rarely consider the possibility that, at a given locus, multiple genes may impact a phenotype by interactions between more than one regulatory allele in across a gene‐haplotype region (Corradin *et al*., 2014). Particularly for complex, multigenic traits such as chlorophyll content, a spatial proximity of interacting genes can potentially facilitate coordinated expression in certain tissues, developmental timepoints or in response to environmental stimuli. This may help explain why multigenic haplogroups associate more strongly with CCI phenotypes than single‐gene haplotypes. The results of the present study underline the benefit of combining haplotype diversity analysis with GWAS studies to dissect additive effects of quantitative trait loci in crops and understand their underlying biology (Buntjer *et al*., 2005). Detailed investigations of trait relationships at the local haplotype level, using high‐density SNP markers in large populations, also provide plant breeders with a means to distinguish between genetic and pleiotropic trait correlations.

## Materials and methods

### Plant materials, genotype and phenotype data

A diversity panel of 203 homozygous *B. napus* inbred lines was constructed to broadly represent variability in Chinese semi‐winter rapeseed, an intermediate form of oilseed *B. napus* that is broadly grown in China and also commonly used to enrich gene pools of European winter oilseed rape and Australian or North American spring canola. The plant population (Table S1) and high‐density SNP data, generated by genotyping with the *Brassica* 60k SNP Illumina consortium genotyping array (Illumina, San Diego, CA), were described in detail by Qian *et al*. (2014).

The association panel was evaluated in separate glasshouse and field experiments. A CCI was calculated based on absorbance measurements at 653 and 931 nm with a CCM‐200 chlorophyll content metre (Opti‐Sciences, Inc., Hudson, NH). Measurements of leaf CCI were performed at seedling and bolting stage in the glasshouse experiment in 2012. At each developmental stage, two independent measurements were taken from each side of a single young leaf on three individual plants per accession. In the field, the accessions were sown in single rows with two replications. Chlorophyll content index measurements were performed on five plants per accession per replication at seedling stage, bolting stage, flowering stage and maturity, using the same measurement procedure as in the glasshouse experiment. Additional field trials for seed quality analysis were performed at the experimental farm of Southwest University in Beibei, Chongqing, China, in 2012 and 2013. Glucosinolate content in harvested, fully mature seeds from all 203 accessions was measured by near‐infrared spectroscopy on seeds grown from all three field environments, recording mean values in glucosinolate (μmol) per seed dry weight (g) from at least two technical and two biological replicates per accession and environment.

### Statistical analysis

Heritability (*H* ^2^) for the two traits was calculated using the statistical software package spss Statistics for Windows Version 22.0 (IBM Corp., Armonk, NY). Distributions and correlations among the traits and environments were analysed using the r package *psych* (Revelle, 2014) and hmisc (Harrell and Dupont, 2012).

### Genome‐wide association analysis

A total of 24 338 high‐quality, single‐locus single‐nucleotide polymorphism (SNP) markers with minor allele frequency (MAF) ≥0.05 were used for the GWAS and LD analyses. The mixed linear model was as follows:

y=Xα+Pβ+Kμ+e

It was used to test associations between the SNPs and phenotypes, where *y* is the vector of phenotypic observations, α is the vector of SNP effects, β is the vector of population structure effects, μ is the vector of kinship background effects, *e* is the vector of residual effects, *P* is the PCA matrix relating *y* to β, and *X* and *K* are incidence matrices of 1s and 0s relating *y* to α and μ, respectively (Yu *et al*., 2006). The observed *P* values from marker‐trait associations were used to display Q–Q plots and Manhattan plots, using r. Kinship analysis was performed using the software TASSEL 5.0 (Bradbury *et al*., 2007), while detailed information on population structure was imported from the previous analysis described by Qian *et al*. (2014). The critical *P*‐value for assessing the significance of SNP‐trait associations was calculated separately for CCI based on a false discovery rate (FDR; Benjamini and Hochberg, 1995). An FDR <0.05 was used to identify significant associations for CCI at cut‐off values of −log_10_^(*P*)^ = 4. To simplify the procedure, we used the uniform Bonferroni‐corrected thresholds at α = 1 as the cut‐offs, so, the Bonferroni threshold (−log^(1/24338)^ = 4.4) was used to identify significant associations for GSL.

### Phenotypic correlations to haplotype diversity groups

Significant haplotype blocks were identified using the r package *LDheatmap* (Shin *et al*., 2006), with haplotypes being defined across regions of homozygous markers LD (*r* ^2^) > 0.65 between the first and last markers in the block. We use the term haplogroup to refer to groups of individuals carrying a common haplotype across a specific haplotype block. Haplogroups with frequency >0.01 were used for comparative phenotype analysis. A two‐sample *t*‐test (assuming unequal variances) was used to test for significant phenotypic differences between haplogroups with regard to leaf CCI and seed GSL. Haplotype networks were constructed based on the SNPs number of haplotype region using the program tcs1.21 (Clement *et al*., 2000).

### Gene content in homoeologous haplotype blocks

A chromosome‐scale alignment of syntenic haplotype block regions on homoeologous chromosomes A05 (position 17 873 133–17 986 390 bp, *r* ^2^ = 0.74) and C05 (36 453 492–37 145 290 bp, *r* ^2^ = 0.68) was performed using the large‐scale genome synteny tool *symap* version 4.2 (Soderlund *et al*., 2011). All annotated genes within the corresponding haplotype regions were extracted from the *B. napus* Darmor‐bzh reference genome v. 4.2 (Chalhoub *et al*., 2014; accessed from https://genomevolution.org/CoGe/). For verification of the most likely gene functions, we accessed annotations of the closest orthologous *Arabidopsis thaliana* gene by blasting to the Arabidopsis genome database http://www.arabidopsis.org/.

### Population structure analysis

The general population structure of the diversity panel was described previously by Qian *et al*. (2014). Here, we further elucidated the detailed substructure within the largest subpopulations, Q1 and ‘mixed’, by reanalysing with a random selection of 11 910 polymorphic, single‐copy SNPs that have MAF ≥0.05 across these subpopulations. This enabled us to accurately distinguish a small clade of 10 related individuals, with high leaf chlorophyll and low seed glucosinolate content, that form a subcluster (clade) within subpopulation Q1. The r package *SelectionTools* (http://www.uni-giessen.de/population-genetics/downloads) was used to perform a principal component analysis, while an unweighted pair group matrix algorithm tree was calculated by powermarker version 3.25 (Liu and Muse, 2005) and drawn using the software figtree version 1.3.1 (Rambaut, 2009).

## Competing interests

The authors declare no competing interests.

## Authors’ contributions

LQ and RS conceived the study, generated the genome‐wide SNP data and drafted the manuscript. WQ generated the plant population. LQ and WQ performed field phenotyping, while LQ performed glasshouse trials and was responsible for the data analysis and interpretation. All authors read and approved the final manuscript.

## Supporting information


**Figure S1** Manhattan and quantile–quantile plots of MLM showing genome‐wide associations for leaf chlorophyll content index in two different environments (glasshouse and field) in 203 Chinese semi‐winter rapeseed accessions.

**Figure S2** Genome‐wide associations for leaf chlorophyll content index on chromosomes A07, A08, C03 and C08, respectively.

**Figure S3** Boxplots showing phenotypic values for leaf chlorophyll content index in nine and three haplogroups, with frequency >0.01, found in haplotype regions on chromosomes A05 and C05, respectively.

**Figure S4** Comparative analysis of leaf chlorophyll content index (CCI) between groups of accessions carrying combinations of two or three of CCI‐associated haplogroups (group A) and accessions carrying only one CCI‐associated haplogroup (group B).

**Figure S5** Boxplots showing leaf chlorophyll content index and seed GSL content in the three different subgroups.

**Figure S6** Association mapping for seed GSL on chromosome A02 in 203 Chinese semi‐winter rapeseed accessions.

**Table S1** Source, population structure and heritability (leaf chlorophyll content index and seed GSL) in 203 Chinese semi‐winter rapeseed accessions

**Table S2** Detailed description of five haplotype regions significantly associated with leaf chlorophyll content index

**Table S3** Comparative analysis of haplogroups related to leaf chlorophyll content index, along with gene content in homologous haplotype regions on chromosomes A05 and C05

**Table S4** Comparative analysis of six haplogroups related to leaf chlorophyll content index, along with gene information in the A02 haplotype region

**Table S5** Comparative analysis of three haplogroups corresponding to leaf chlorophyll content index in BnaA02g20650D gene region

**Table S6** Comparative analysis of five haplogroups corresponding to leaf chlorophyll content index and seed GSL and gene information in chromosome A01 coselection haplotype region

**Table S7** The distribution of haplogroups related to higher leaf chlorophyll content index accessions and comparative analyses of three clades corresponding to leaf CCI and seed GSL in subpopulations ‘Q1’ and ‘mixed’, respectively

**Table S8** Comparative analysis of leaf chlorophyll content index between A and B groups

**Table S9** Gene information in the A02 haplotype region
