# Supplementary material for: Haplotype hitchhiking promotes trait coselection in Brassica napus
Source: Plant Biotechnol J. 2016 Jan 23;14(7):1578–88. doi: 10.1111/pbi.12521 (PMC5066645; doi:10.1111/pbi.12521)

Figure S1 Manhattan and quantile-quantile (QQ) plots of MLM showing genome-wide associations for leaf CCI in two different environments (greenhouse and field) in 203 Chinese semi-winter rapeseed accessions. The –log10(p) significant threshold of 4 is indicated with a horizontal blue line.
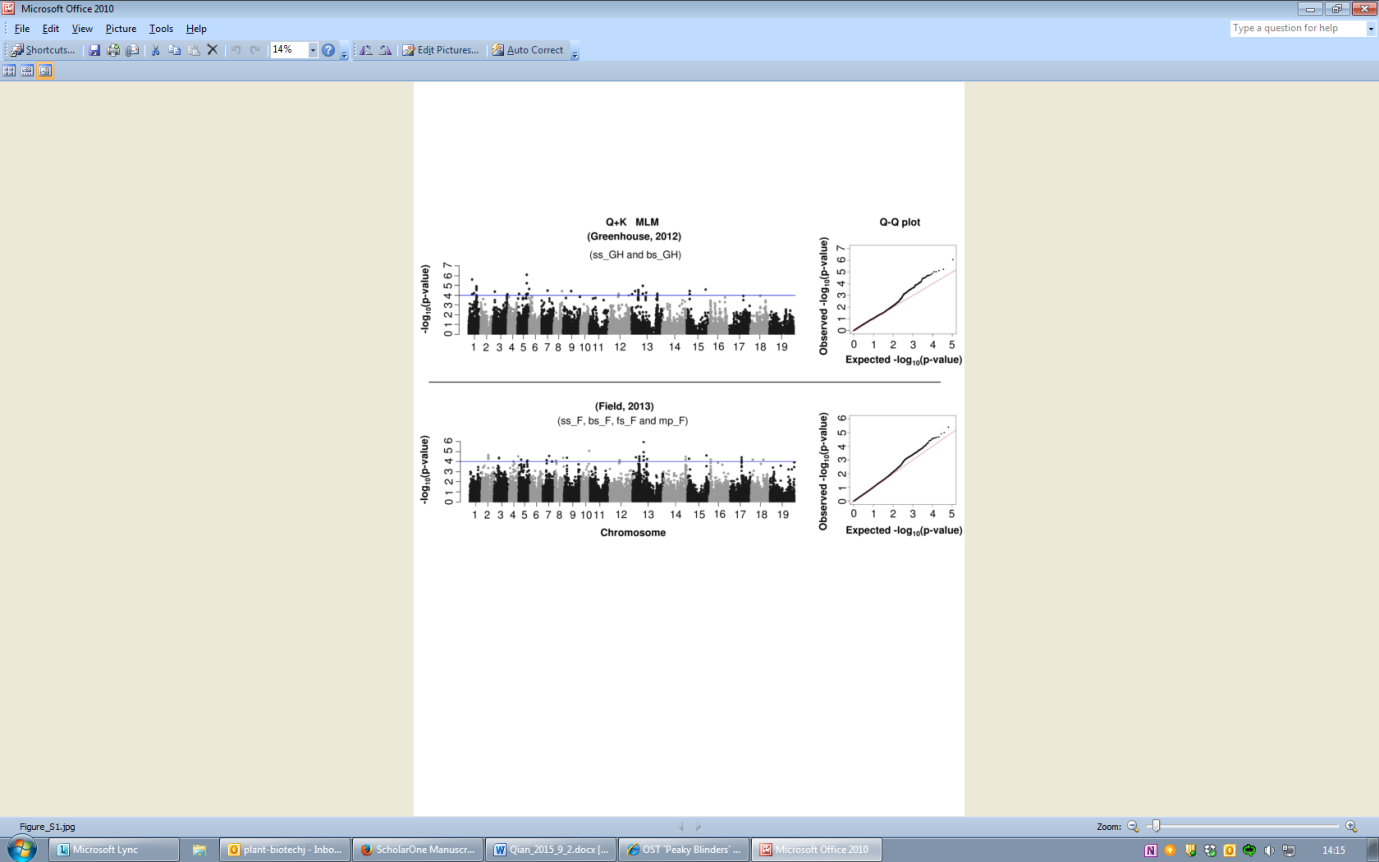

Supplement: Supplementary file 1 — Figure S1 Manhattan and quantile–quantile plots of MLM showing genome‐wide associations for leaf chlorophyll content index in two different environments (glasshouse and field) in 203 Chinese semi‐winter rapeseed accessions. [file PBI-14-1578-s001.docx]
