# Supplementary figures and images for: Haplotype hitchhiking promotes trait coselection in Brassica napus
Source: Plant Biotechnol J. 2016 Jan 23;14(7):1578–88. doi: 10.1111/pbi.12521 (PMC5066645; doi:10.1111/pbi.12521)

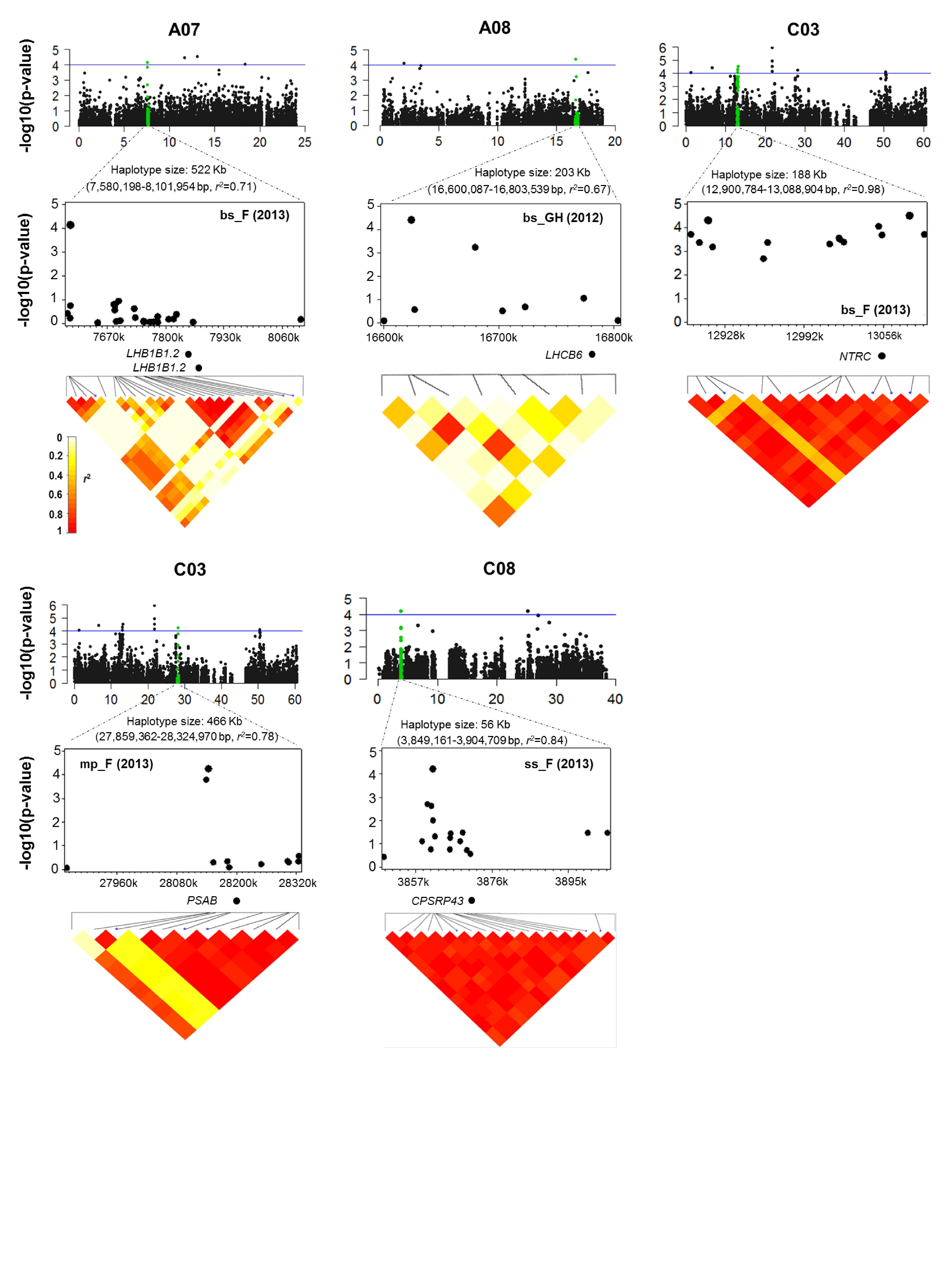

Supplement: Supplementary file 2 — Figure S2 Genome‐wide associations for leaf chlorophyll content index on chromosomes A07, A08, C03 and C08, respectively. [file PBI-14-1578-s013.jpg]

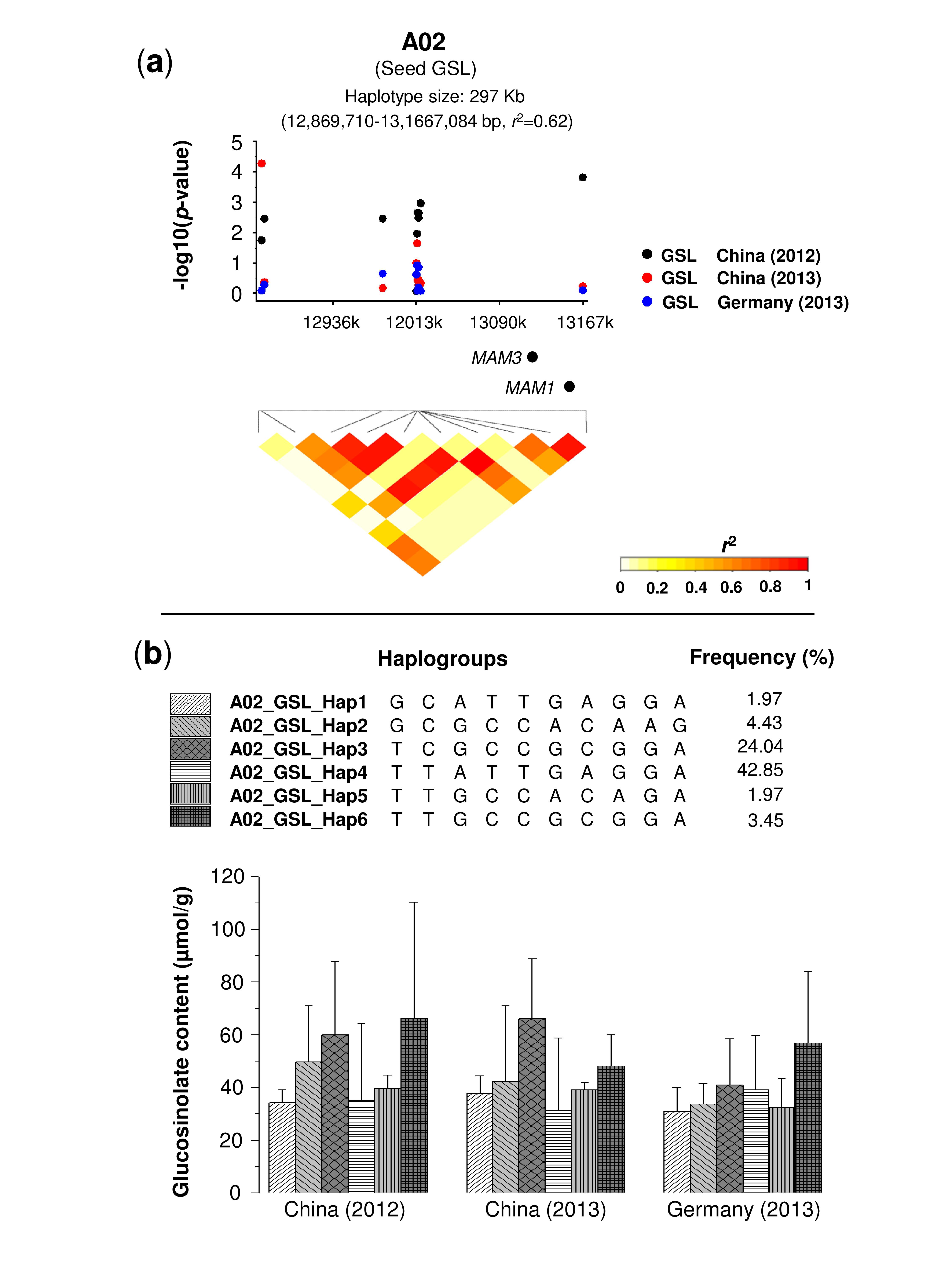

Supplement: Supplementary file 6 — Figure S6 Association mapping for seed GSL on chromosome A02 in 203 Chinese semi‐winter rapeseed accessions. [file PBI-14-1578-s002.jpg]
