# Supplementary material for: Haplotype hitchhiking promotes trait coselection in Brassica napus
Source: Plant Biotechnol J. 2016 Jan 23;14(7):1578–88. doi: 10.1111/pbi.12521 (PMC5066645; doi:10.1111/pbi.12521)
Supplement: Supplementary file 3 — Figure S3 Boxplots showing phenotypic values for leaf chlorophyll content index in nine and three haplogroups, with frequency >0.01, found in haplotype regions on chromosomes A05 and C05, respectively. [file PBI-14-1578-s012.docx]

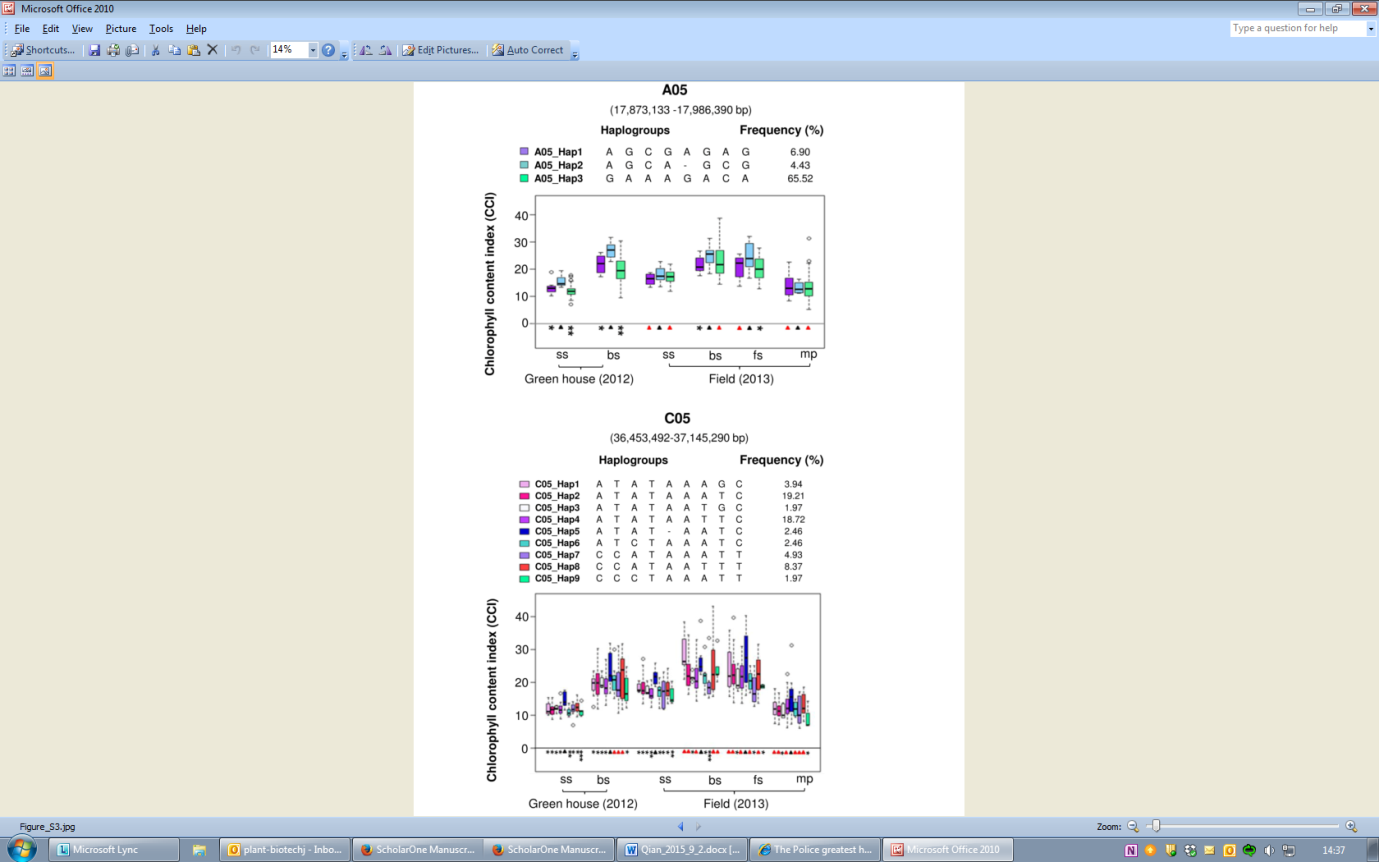


Figure S3 Boxplots showing phenotypic values for leaf CCI in nine and three haplogroups, with frequency greater than 0.01, found in haplotype regions on chromosomes A05 and C05, respectively. Comparative analyses of haplogroups showed that A05_Hap2 and C05_Hap5 have higher leaf CCI than the other haplogroups. Symbols show significant differences of haplogroups compared with A05_Hap2 and C05_Hap5: *p ≤0.05, **p ≤0.01, ***p ≤0.001; Red triangles: not significant (p > 0.05).
