# Supplementary material for: Haplotype hitchhiking promotes trait coselection in Brassica napus
Source: Plant Biotechnol J. 2016 Jan 23;14(7):1578–88. doi: 10.1111/pbi.12521 (PMC5066645; doi:10.1111/pbi.12521)

Figure S4 Comparative analysis of leaf CCI between groups of accessions carrying combinations of two or three of CCI-associated haplogroups (group A)and accessions carrying only one CCI-associated haplogroup (group B).
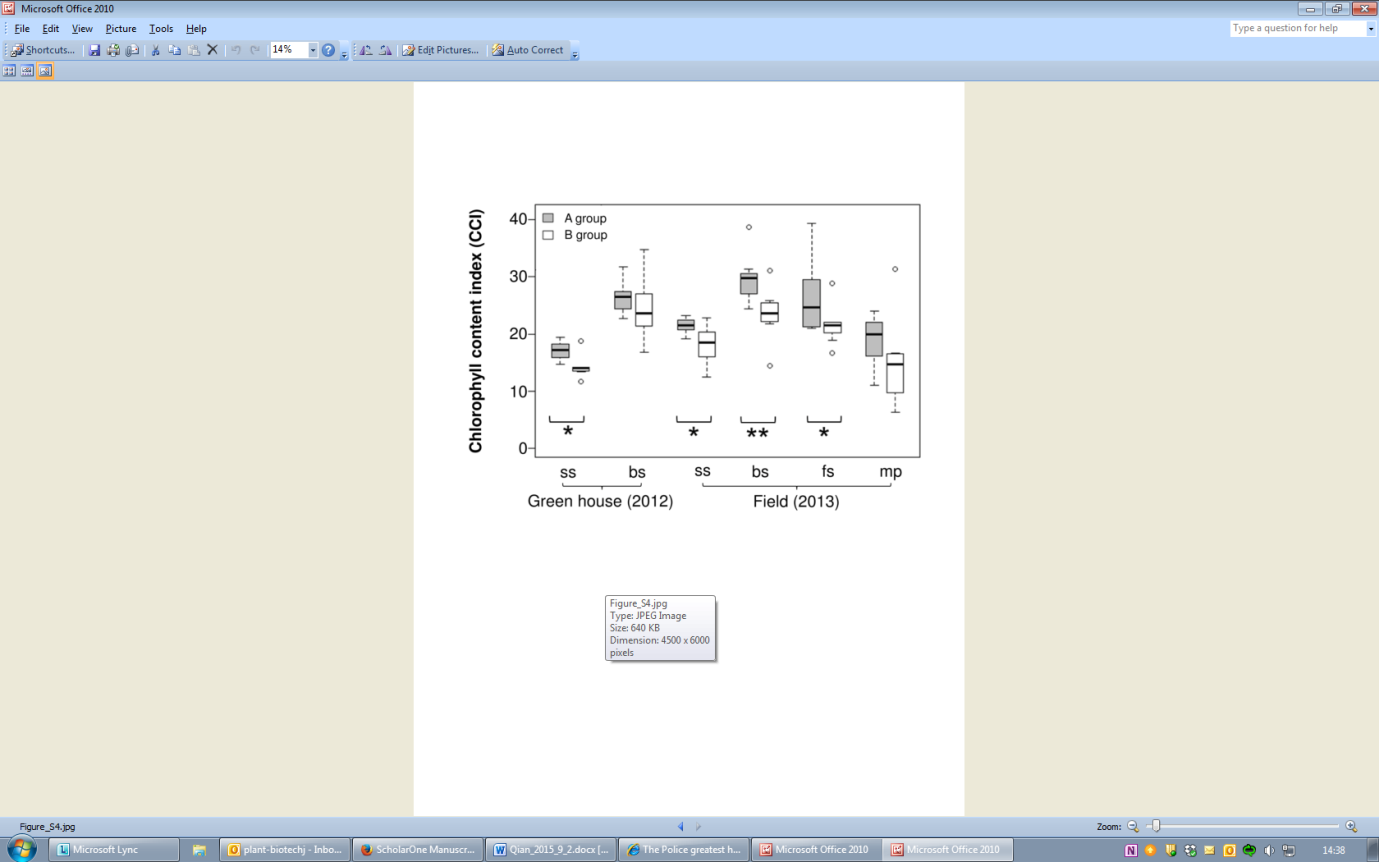

Supplement: Supplementary file 4 — Figure S4 Comparative analysis of leaf chlorophyll content index (CCI) between groups of accessions carrying combinations of two or three of CCI‐associated haplogroups (group A) and accessions carrying only one CCI‐associated haplogroup (group B). [file PBI-14-1578-s014.docx]
