# Supplementary material for: Haplotype hitchhiking promotes trait coselection in Brassica napus
Source: Plant Biotechnol J. 2016 Jan 23;14(7):1578–88. doi: 10.1111/pbi.12521 (PMC5066645; doi:10.1111/pbi.12521)

Figure S5 Boxplots showing leaf CCI and seed GSL content in the three different subgroups. Accessions belonging to Q1_2 always show elevated leaf CCI and lower seed GSL. Symbols show significant differences of haplogroups compared with Q1_2: *p ≤0.05, **p ≤0.01, ***p ≤0.001; Red triangles: not significant (p > 0.05).
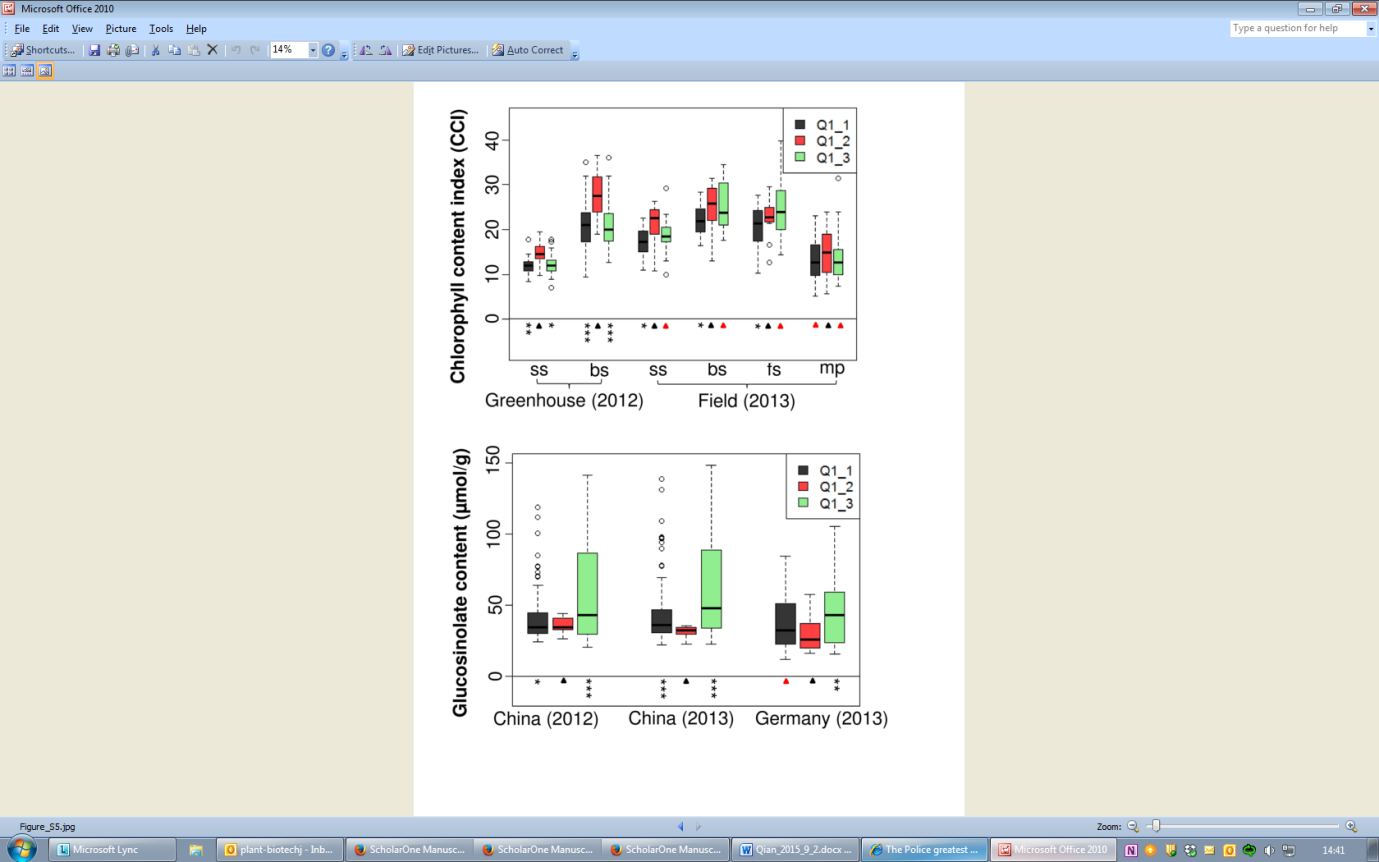

Supplement: Supplementary file 5 — Figure S5 Boxplots showing leaf chlorophyll content index and seed GSL content in the three different subgroups. [file PBI-14-1578-s015.docx]
